# Standards Compliance and Health Implications of Bottled Water in Malawi

**DOI:** 10.3390/ijerph16060951

**Published:** 2019-03-16

**Authors:** Russel C. G. Chidya, Lazarus Singano, Isaac Chitedze, Khaldoon A. Mourad

**Affiliations:** 1 Faculty of Environmental Sciences, Department of Water Resources Management and Development, Mzuzu University, P/Bag 201 Mzuzu, Malawi; 2 Department of Agricultural Research Services, Ministry of Agriculture, Irrigation and Water Development, Chitedze Agricultural Research Station, P.O. Box 158 Lilongwe, Malawi; lasingano@yahoo.com; 3 Faculty of Science, Technology and Innovations, Department of Energy Systems, Mzuzu University, P/Bag 201 Mzuzu Malawi; isaacchitedze@gmail.com; 4 Faculty of Social Sciences, Centre for Middle Eastern Studies, Lund University, 22100 Lund, Sweden; khaldoon.mourad@cme.lu.se

**Keywords:** physico-chemical, microbiological, water quality, food safety, Lilongwe, Malawi

## Abstract

Many people around the globe prefer bottled water especially in developing countries, where tap water is not drinkable. This study investigated the quality of bottled drinking water sold in Lilongwe city, Malawi. Compliance with Malawi Standards (MS) 560 (2004) for natural mineral water, MS 699 (2004) for bottled water and the World Health Organisation guidelines for drinking water were examined. Bottled water from different 12 brands was purchased from local stores and analysed for its pH, total dissolved solids (TDS), EC, turbidity, Ca, Mg, Na, K, Fe, NO_3_^−^, Cl^−^, F^−^, SO_4_^2−^, hardness, alkalinity, and *Escherichia coli*. A Hierarchical Cluster Analysis (HCA) resulted in two clusters in which most of the brands (92%, *n* = 12) belonged to one group. The two clusters and significant differences (ANOVA *p* < 0.05) in chemical compositions among the brands were attributed to the variations in the water source and the treatment processes. The results showed that 10 brands did not comply with the MS 699 (2004) turbidity standard (1 NTU) and the pH of one of the brands was below the minimum MS 699 (2004) standard of 6.50. This research showed that 12 brands had bottle labelling errors and discrepancies in chemical composition. The article highlighted the need for a strict inspection from the responsible governmental ministry to improve water quality and to adjust water bottles’ labels according to water characteristics.

## 1. Introduction

Water is a finite resource that plays a fundamental role in food security, economic growth, energy production, and public health among others [1,2]. Clean water improves human life, predominantly in the deterrence of the spread of disease-causing microorganisms [3,4]. Provision of clean and safe water is undeniably paramount to human health, hence given priority concern by the United Nations (UN) Sustainable Development Goal (SDG) number 6. This goal, among other targets, is set to achieve universal and equitable access to safe and affordable drinking water for all by 2030 [4,5].

An increasing number of countries are confronting water stress that affects more than two billion people globally. Achievement of the SDG 6, therefore, requires concerted efforts by all stakeholders [4]. In line with such efforts, several policy frameworks, instruments and legal documents have been instituted by the Malawi government to address and improve the water, sanitation and hygiene (WASH) sectors. These include Malawi Vision 2020, National Water Policy (2005) and the recently launched Malawi Growth and Development Strategy (MGDS) III (2017–2022) that recognizes the importance of safe drinking water, sanitation and integrated water resources management (IWRM). Further, the Water Works Act (1995) regulates water supply and sanitation services in urban areas of Malawi and defines operation of its five water boards, namely Lilongwe, Blantyre, Central, Southern and Northern Regions. Access to safe and potable water in the country is mainly provided via taps in cities and urban areas, while rural areas are mostly supplied using boreholes [6].

In recent years, bottled drinking water has emerged as an easy way through which potable water is supplied to consumers, especially in cities and urban areas [7]. In many developing countries including Malawi, the market for bottled drinking water, mineral and natural spring waters is increasing. The dramatic rise in the consumption of bottled water globally is attributed to its affordability, convenience, consumer connotations of higher social status and the prevalent conception that it contains fewer contaminants [8,9]. Furthermore, there is a common belief and perception that mineral waters have valuable medicinal and healing effects. Bottled drinking water provides an essential mineral supplement for proper growth and has also found widespread usage in infant formula preparation, re-forming other foods, filling humidifiers and skin care [10,11]. The bottled drinking water is also perceived to taste better compared to tap water and is served at many organized gatherings, such as meetings, parties, workshops, and conferences [12,13]. In some countries, the consumption of bottled water reflects health concerns surrounding the safety of public water supplies. Based on the water sources and treatment processes, bottled water is generally categorised into mineral, spring, purified (still) and carbonated water [9,14].

Despite the increase in consumption of bottled drinking water worldwide, it is becoming an important factor both for economic and human health that requires strict standardization and periodic inspection. The World Health Organisation (WHO) provides general guidelines for drinking water quality [4]. In Malawi, the Malawi Bureau of Standards (MBS) formulates national standards for drinking water and other fields of interest. For instance, the Malawi Standards (MS) 560 (2004) and MS 699 (2004) provides specifications for natural mineral waters and bottled drinking waters other than natural mineral water [14,15]. Studies elsewhere have shown non-compliance and irregularities in the analytical and chemical composition of the bottled drinking water. Furthermore, studies elsewhere have shown contamination and dubious labelling of bottled drinking water [9,16,17,18]. Bottle labelling of the chemical composition is of concern and, for example, cases of undervaluing and exaggeration have been reported elsewhere [16,17,18]. Despite an increase in consumption of bottled drinking water in Malawi, independent study reports on their chemical composition, safety and compliance with standards are not readily available in literature. To the best of our knowledge, there are either scanty or no published reports in literature on chemical composition of the bottled drinking water sold in various cities and towns of Malawi. This study, therefore, investigated the chemical composition of bottled drinking water sold in Lilongwe city, Malawi. The study primarily focused on the physico-chemical and microbiological water quality, their compliance with national and international drinking water standards and potential health implications associated with consumption of the bottled drinking water.

## 2. Materials and Methods

### 2.1. Description of the Study Area

**Figure 1:**
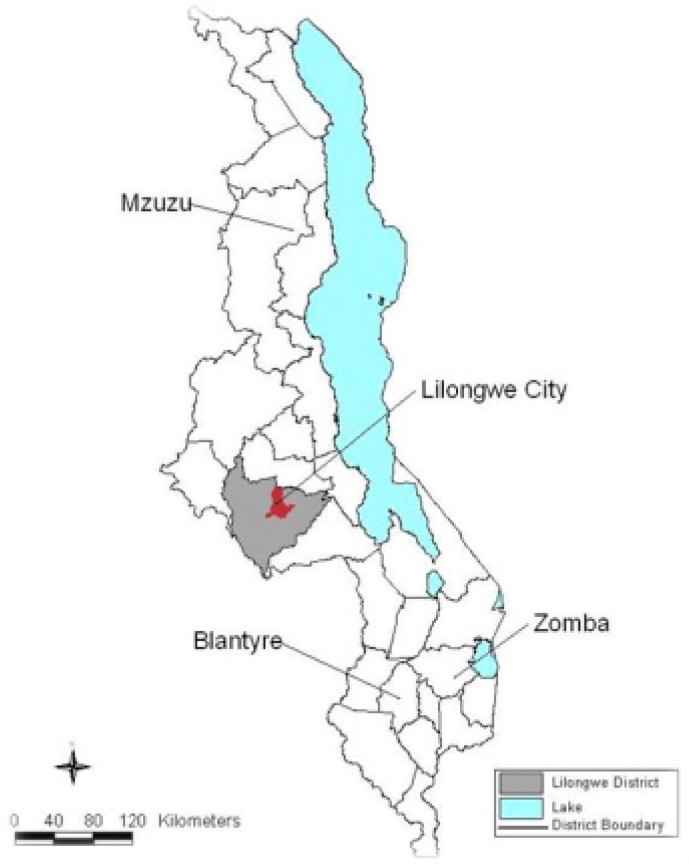
Map of Malawi showing the location of Lilongwe and other cities.

The study was conducted in Lilongwe city located in the central region of Malawi (Figure 1). The city lies between 33.5 E–34.5 E longitudes and 14.5 S–13.5 S latitudes at an altitude of 1050 m above sea level. The total population of Lilongwe city reached 989,318 in 2018 [19]. Lilongwe is bordered by four districts, namely Dedza, Salima, Mchinji and Dowa. It is also bordered by the Republic of Mozambique and Zambia. The district generally features a humid sub-tropical climate that borders on a subtropical highland climate, with warm summers and mild winters. The mean annual temperature ranges from 20 °C to 25 °C. The district experiences a short-wet season that runs from December to March and a lengthy dry season that covers much of the remaining months. The area usually receives heavy downpours and an annual average rainfall of 800–900 mm [20,21].

Lilongwe has been experiencing rapid urbanization and is viewed as the fastest growing city in the country. The booming social and economic developments have led to a population growth from 19,425 in 1966 to 674,448 in 2018 and 989,318, with an estimated annual growth rate of 3.8% and population density of 2453 persons per km^2^ [19,20,21,22]. The city provides a significant business, economic and transportation hub for central Malawi. Several government and private offices, small to large businesses, retail shops and malls are in the new city centre and peripheral of the city. These offices and shops are supplied with tap water from Lilongwe Water Board (LWB) and bottled drinking water from various private companies. The water board is reported to serve about 63% of the city population with tap water, mostly through individual household connections and kiosks run by Water Users Associations (WUAs), Kiosk Management Unit (KMU) and private operators [5,21,22,23,24]. The LWB gets its water from upstream of Lilongwe river at Malingunde (Kamuzu Dam 1 and 2). Apart from taps and bottled drinking water, people in the city also rely on other water sources, such as boreholes, which are sunk in some peri-urban and low-income areas [5,23].

### 2.2. Sample Collection and Bottled Water Database

Bottled drinking water from 12 commercial brands was purchased from simple randomly selected supermarkets and retail shops in Lilongwe City. For each brand, 10 separate samples were collected in triplicate from those supermarkets and retails shops. The number of brands available on the market during the sampling period determined the total sample size (*n* = 360). The bottled drinking water of 500 mL volume had a validity of one year, and all sampled bottles were within the safe period. Standard operating procedures and protocols were followed to collect, preserve and transport the water samples to avoid any cross contamination and alteration of the samples: MS 682-1:2002, MS 682-3:2002 [25,26,27]. Immediately after collection, all the samples were transported to Central Government Water Laboratory of the Ministry of Agriculture, Irrigation and Water Development (Malawi) for analysis. Prior to analyses, all the laboratory and field equipment were washed thoroughly with distilled water and handled following standard procedures [27]. Apparatus for microbiological analysis were sterilised prior to their use. 

The names of the water brands (*n* = 12) sampled were: “Aquamist, Aqua-Pure, Mkokomo, Zabo, Pure Zone, Nestle Pure Life, P-water, Premium Still Water (Quench), Hayat, AquaA^+^, Nyika, and Real”. The chemical compositions reported on the bottle labels were collected for checking compliance with standards and our results. For ethical considerations, real names of the brands were not used instead coded B1 to B12 (not in the order of appearance of the brand names). A summary of information on water type, source, treatment methods and the number of water quality parameters reported on bottle labels is presented in Table 1. The reference water quality parameters (*n* = 13) used for this comparison were pH, TDS, turbidity, alkalinity (as CaCO_3_), Ca, Mg, Na, K, Fe, Cl^−^, SO_4_^2−^, NO_3_^−^, and F^−^.

Most of the brands used in the study were of purified still (uncarbonated) water, while two brands (B1 and B2) had their water sources from spring and natural mineral. Carbonated water was not included in this study as it was not available on the market at the time of the study. On the one hand, ‘*natural mineral water*’ is defined by Directive 2009/54/EC as microbiologically wholesome water originating in an underground water table or deposit and emerging from a spring tapped at one or more natural or bore exits. It may not be subjected to any treatment apart from the separation or introduction of constituents. On the other hand, ‘*spring water*’ is defined as water derived from a subterranean source from which water flows naturally to the surface of the earth [14,28].

### 2.3. Analytical Methods and Water Quality Analyses

All reagents and chemicals used in the analyses were of analytical grade, sourced from British Drug House (BDH), Merck in Germany and South Africa. Distilled water was used in all dilutions, rinsing of apparatus and preparation of standard solutions. The study employed standard analytical methods and the following physico-chemical and microbiological water quality parameters were measured: pH, total dissolved solids (TDS), electrical conductivity (EC), turbidity, calcium (Ca), magnesium (Mg), sodium (Na), potassium (K), iron (Fe), nitrate (NO_3_^−^), chloride (Cl^−^), fluoride (F^−^), sulphate (SO_4_^2−^), hardness, alkalinity and faecal coliform bacteria.

#### 2.3.1. Physico-Chemical Water Quality Parameters

The pH, TDS, EC and turbidity: A pH meter (EUTECH Instruments pH/mv/C/F meter), pre-calibrated with buffer solutions (pH 4 and 7) was used to measure the pH values of the water samples. The TDS (mg/L) and EC (µS/cm) were determined by Wagtech Conductivity meter. Prior to TDS/EC measurements, the meter was calibrated with standard potassium chloride (KCl) solution. Further, the electrode of the meter was rinsed thrice with distilled water and wiped with clean tissue paper after each measurement to avoid cross-contamination. Turbidity in nephelometric turbidity units (*NTU*) was measured by a digital turbidimeter (HF INSTRUMENTS, DRT 100) calibrated by a 0.14 standard solution.

NO_3_^−^, F^−^, SO_4_^2−^, Cl^−^, and alkalinity: NO_3_^−^ were determined by spectrometric method using sulfosalicylic acid [29,30,31]. Standard NO_3_^−^ solutions (0.01, 0.02, 0.04, 0.08, 0.10 and 0.20 mg/L NO_3_^−^) were prepared according to the standard procedures and appropriate sample dilutions were made to analyse samples, of which concentration was greater than 0.2 mg/L. The nitrate analyses were performed with the aid of a UV-Vis spectrophotometer (HACH DR/3000) at 410 nm wavelength. Sulphate was determined by turbidimetric method with the aid of a UV-Vis spectrophotometer (DR/3000) [27]. The method is based on precipitation of sulphate ions as insoluble barium sulphates presented in Equation (1) [27,32].

(1) $$SO_{4}^{2-}\left(aq\right)+Ba^{2+}\left(aq\right)\to BaSO_{4}\left(s\right)$$

The sulphate standard solutions and water samples were measured with the UV-spectrophotometer at 420 nm wavelength. Fluoride was determined using SPADNS spectrophotometric method [27]. In this method, fluoride ions were reacted with zirconium dyes, Zr-SPADNS (sodium 2-(parasulphophenylazo)-1,8-dihydroxy-3,6-naphthalene disulphonate). After colour development, the absorbance of the resulting coloured complex was then measured using a UV-Vis spectrophotometer (HACH DR/3000) at 570 nm wavelength. Chloride was determined by titration (Mohr) method [33]. The water samples were titrated against a standard solution of silver nitrate using potassium chromate as an indicator, and concentrations were calculated accordingly. Alkalinity (as carbonates and bicarbonates in mg/L CaCO_3_) was determined by titration method with sulfuric acid (H_2_SO_4_) using phenolphthalein and methyl orange indicators [27].

Ca, Mg, Na, K and Fe: The sodium and potassium were measured using flame photometry with the aid of a flame photometer (Corning, Model 400). Fe was measured by a sequential inductively coupled plasma atomic emission spectrometer (ICP-AES). The calcium and magnesium were determined by EDTA-titrimetric method [27,34]. Hardness is most commonly expressed as milligrams of calcium carbonate equivalent per litre. The total water hardness, as Ca^2+^ and Mg^2+^ ions, was calculated according to Equation (2) [35,36]:

(2) Total hardness=2.5 [Ca]+4.1 [Mg]

where [Ca] and [Mg] are the calcium and magnesium concentrations (in mg/L) measured in the water samples, while 2.5 and 4.1 are their molar mass ratios per 100 g CaCO_3_.

#### 2.3.2. Microbiological Water Quality Analysis

Faecal coliforms (FC) are bacteria that originate specifically from the intestinal tract of warm-blooded animals. Their occurrence in any water source gives an indication of faecal contamination and the presence of disease-causing microorganisms [37]. In our study, possible contamination of bottled drinking water with microorganisms was assessed by testing for the presence of *Escherichia coli* using standard procedures [38,39]. The *E. coli* bacteria were analysed using membrane filtration method (MFM) technique. The analyses were performed within a 24-h required period after sampling. The bottled water sample (100 mL) was filtered through a special membrane (47 mm diameter, 0.20 µm pore size) which retains the bacteria. Petri dishes containing filter membranes and selective nutrient media (M-Lauryl Sulphate Broth) for *E. coli* were incubated at 44.5 ± 0.5 °C for 24 h. The number of bacteria colonies were expressed as colony-forming units (cfu) per 100 mL. Physical counting of bacteria colonies was done with the aid of a colony counter (Bibby Stuart Scientific Ltd., Stone, UK). Representations of colonies formed from each plate were picked for microscopy tests to confirm their identity [38].

### 2.4. Data Management and Statistical Analysis

To ensure quality control and reliability of the data, samples were collected in triplicates and all procedures for both sample collection and analysis were made following standard methods [14,15,27]. Method blank and control samples were also prepared and analysed to ensure quality control, to improve the reliability of chemical measurements and to increase confidence in the experimental data. Furthermore, sample analyses were made at a reputable institution where equipment and staff are certified. Descriptive statistics, such as minimum, maximum, averages and ranges, were performed using Microsoft Excel (2016). Statistical Package for the Social Sciences (SPSS version 20) was employed for multivariate statistical analysis, namely Hierarchical Cluster Analysis (HCA) and Principal Component Analysis (PCA). The Kaiser–Meyer–Olkin (KMO) and Bartlett’s test were used to verify the suitability of data sets before running PCA Varimax rotated with Kaiser normalization. The HCA and PCA were employed to characterise associations present in the chemical composition data sets. Significant differences of parameters amongst brands and standard values were calculated by one-way analysis of variance (ANOVA, *p* < 0.05). Correlation analysis (Pearson’s correlation r, *p* = 0.05 and *p* = 0.01) was employed to evaluate the statistical significance and the relationships amongst water quality parameters and the bottled water brands.

### 2.5. Ethical Approval

The study was approved by the director of research at Mzuzu University, Malawi in line with ‘General Conduct of Research and Ethical Consideration’ and Mzuzu University Research Code of Conduct.

## 3. Results and Discussion

### 3.1. Physico-Chemical Water Quality and Their Health Implications

**Figure 2:**
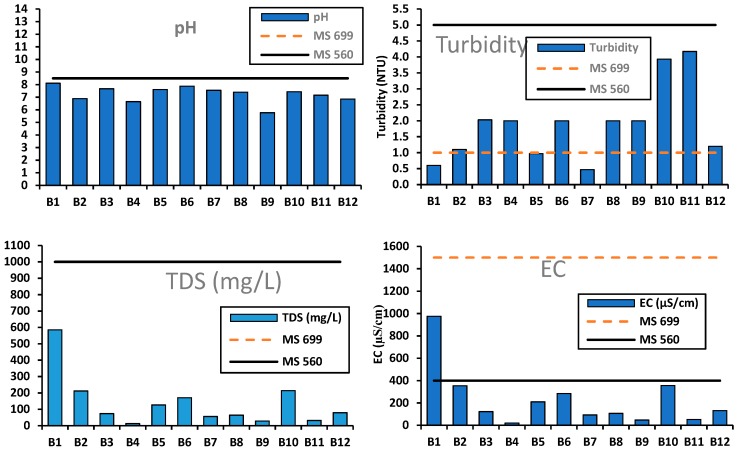
Variations in water quality (pH, turbidity, EC and TDS) of the bottled water compared to MS 699 (treated bottled water) and MS 560 (natural mineral water) standards. Data for each brand is the mean values of the total samples (*n* = 30).

**Figure 3:**
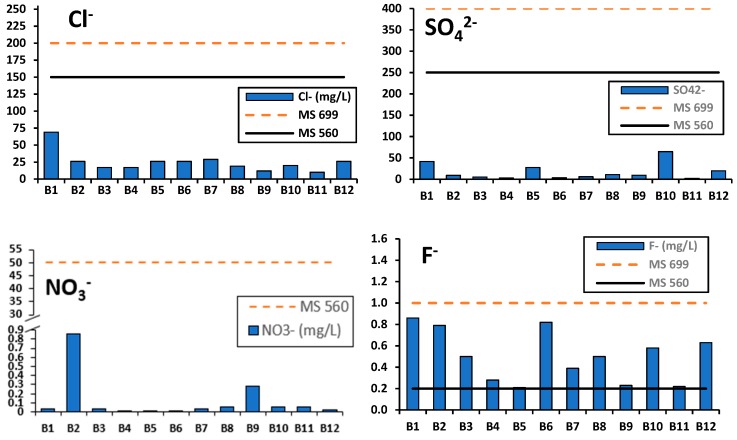
Variations in major anions (Cl^−^, SO_4_^2−^, NO_3_^−^, and F^−^) in mg/L compared to MS 699 (bottled water) and MS 560 (natural mineral water) standards. Data for each brand is the mean values of the total samples (*n* = 30).

**Figure 4:**
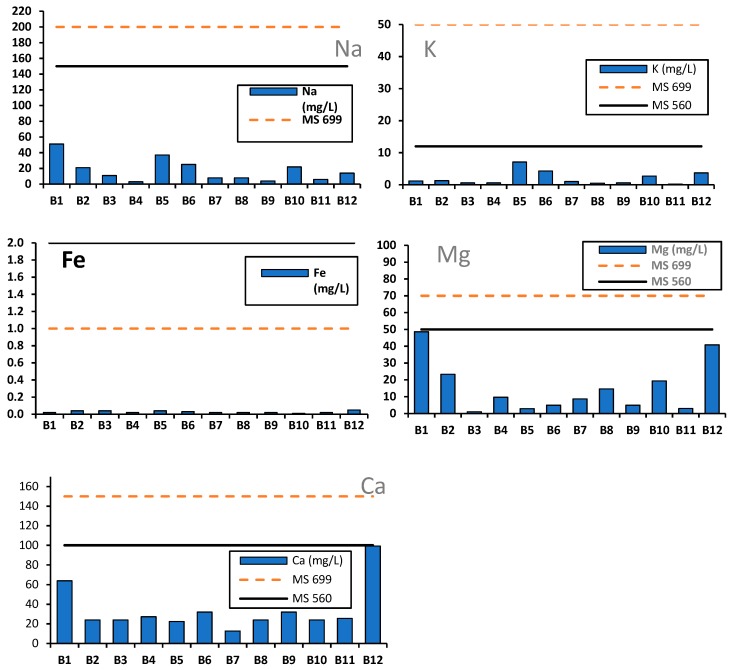
Variations in mineral elements (Na, K, Fe, Ca and Mg) in mg/L for the 12 brands compared to MS 699 (bottled water) and MS 560 (natural mineral water) standards. Data for each brand is the mean values of the total samples (*n* = 30).

Figure 2, Figure 3 and Figure 4 show results on the physico-chemical water quality compared and plotted against the Malawi Standards (MS) 560 (2004) for natural mineral water specifications and MS 699 (2004) for bottled waters other than natural mineral waters. Further, the WHO guidelines for drinking water quality were also conferred [14,15,37]. The results generally showed that the bottled water brands had different characteristics and chemical compositions attributed to several factors, including differences in treatment processes, water source, chemical analysis processes, and dubious bottle labelling.

The pH, TDS, EC, turbidity: The variations in pH, turbidity, TDS and EC amongst the brands compared to the Malawi standards are shown in Figure 2. The pH of brands B3–B12 complied with the maximum MS 699 (2004) pH guideline value of 8.5. The B1 and B2, being natural mineral water brands, also complied with the maximum MS 560 (2004) pH guideline value of 8.5. However, one brand (B9) had its mean pH (5.77) below the minimum MS 699 (2004) guideline value of 6.5. Although no health-based guideline value is projected for pH due to its insignificant health concerns in drinking water [37], continued consumption of water from brand S9 may pose a health hazard to consumers by damaging certain body tissues.

Turbidity ranged from 0.47 to 4.17 NTU and most of the brands for treated bottled water (B3, B4, B6, B8–B12) (80%, *n* = 10) scored values above the MS 699 (2004) guideline of 1 NTU (Figure 2). Although many of these brands apparently treated their waters, it is evident that the processes were not effective enough to remove suspended solids and coagulated particles responsible for turbidity in water. Two brands, namely B1 (0.60 NTU) and B2 (1.10 NTU), complied with natural mineral water turbidity guideline value of 5 NTU set by MS 560 (2004). Despite these two brands complying with the set standards, their turbidity levels were significantly different (ANOVA, *p* < 0.05) from the rest of the brands. This was attributed to their sources of water as well as treatment processes. Although turbidity has inconsistent health significance at levels found in drinking water [37], it is a good indicator of the presence of hazardous chemical and microbial contaminants, hence many consumers tend to associate turbidity with safety. A study on a review of epidemiological studies of the association between turbidity of drinking-water supplies and incidence of acute gastrointestinal illness (AGI) reported positive associations between drinking-water turbidity and AGI incidence with both unfiltered and filtered supplies [40]. An association between turbidity and gastrointestinal illness exists in some settings or over a certain range of turbidity has also been reported in the literature [41]. Consequently, the higher turbidity levels observed in this study in the two brands should be of concern in as far as human health is concerned.

The mean TDS (12.34–585.4 mg/L) and EC (20.57–975.67 µS/cm) in all the brands complied with both MS 699 (2004) and MS 560 (2004) (Figure 2). The palatability of water with a TDS < 600 mg/L is generally considered to be healthy, however, the drinking-water may become expressively unpalatable at TDS > 1000 mg/L [37]. In this study brand B1, with TDS 585 mg/L, was bound to be fairly unpalatable. Significant differences (ANOVA, *p* < 0.05) existed amongst brands for both TDS and EC.

Cl^−^, SO_4_^2−^, NO_3_^−^, and F^−^: The analytical results on chloride (10–69 mg/L) showed that all the brands complied with the MS 699 (2004) and MS 560 (2004) guideline values of 200 and 150 mg/L, respectively (Figure 3). WHO [37] has no guideline value for chloride considering its insignificant levels found in drinking water. However, excess chloride (>250 mg/L) in drinking water can give rise to noticeable taste [37]. All the brands at the time of the study were therefore expected to have good taste since the chloride levels were well below 250 mg/L. Despite complying with the MS, significant differences (ANOVA, *p* < 0.05) existed amongst the brands for chloride. Similarly, sulphate (2.06–64.69 mg/L) complied with both the MS 699 (2004) and MS 560 (2004) guideline values of 400 and 250 mg/L, respectively. Sulphate ions greater than 400 mg/L markedly damage the potability of drinking water [37,42]. Consequently, all the bottled drinking water brands were found to be potable, palatable and safe for consumption in line with sulphate. Significant differences (ANOVA, *p* < 0.05) existed amongst brands in which B10 registered the highest sulphate concentrations (64.70 mg/L).

Nitrate ranged from 0.01 to 0.86 mg/L, and Brands B1 and B2 complied with the MS 560 (2004). There is no guideline value set for NO_3_^−^ by MS 699 (2004) due to its purportedly insignificant effects at levels found in drinking water. However, the NO_3_^−^ guideline and short exposure taste threshold value of 50 mg/L is set by WHO to protect against methemoglobinemia and thyroid effects in bottle-fed infants and the most prone subpopulation, and subgroups [37,42]. In this study, all the brands complied with WHO NO_3_^−^ taste threshold values such that cases of methemoglobinemia and thyroid effects could not be attributed to use of the bottled water.

Fluoride ranged from 0.21 to 0.86 mg/L (Figure 3) in all the brands. The Malawi standard (MS 560: 2004) instituted a specification of 0.2 mg/L at 25 °C for fluoride in natural mineral waters. In this study, the mineral water brands B1 (0.86 mg/L) and B2 (0.79 mg/L) failed to comply with this fluoride specification. Although all the brands complied with the MS 699 (2004) for fluoride (1 mg/L) in drinking water, some few brands (B1: 0.86 mg/L, B2: 0.79 mg/L, and B6 0.82 mg/L) showed fairly higher values, hence the may pose a dental health hazard to consumers. Excessive uptake of fluoride via drinking water is reported to cause mild dental fluorosis [37]. The WHO set a guideline value of 1.5 mg/L fluoride in drinking water and artificial fluoridation in the range of 0.5–1.0 mg/L is recommended for drinking waters with very low fluoride content. In this study all the brands complied with the WHO 1.5 mg/L F^−^ guideline value. However, the four brands B4, B5, B9 and B11 which had lowest fluoride concentrations (<0.28 mg/L) would require artificial fluoridation to prevent dental caries in consumers. The alkalinity ranged from 20 to 320 mg/L CaCO_3_ in which the highest was recorded in brand B12. There are no guideline values set for alkalinity by the MS 560 (2004), MS 699 (2004) and WHO for natural mineral water, bottled drinking water and general drinking water quality, respectively. Lower alkalinity values (16–79 mg/L CaCO_3_) compared to our study have been reported in Mwanza City in Tanzania [17].

Na, K, Fe, Ca and Mg: The mean concentration ranges for *Na* and *K* were 3–51 mg/L and 0.2–7.1 mg/L, respectively (Figure 4). Brands B3–B12 scored *Na* concentrations below the MS 699 (2004) guideline value of 200 mg/L. Similarly, the natural mineral water brands B1 and B2 scored sodium below the MS 560 (2004) standard of 150 mg/L. However, B1 showed fairly higher sodium (51 mg/L) compared to all the brands, while brand B4 had the lowest (3 mg/L). Comparably, all the brands complied with both the MS 699 (2004) and MS 560 (2004) potassium standard values of 50 and 12 mg/L for bottled drinking water and natural mineral waters, respectively. The mean values for both *Na* and *K* were significantly different (ANOVA, *p* < 0.05) amongst brands. Similar *Na* (0.1–48 mg/L) and *K* (0.1–7.0 mg/L) concentrations have been reported in 73 bottled water44 brands from Iran (Kermanshahi et al., 2010). Furthermore, similar findings were reported for *Na* (12–15.4 mg/L) and *K* (4.6–7.12 mg/L) in bottled water in Bolgatanga municipality, Ghana [43].

Although iron may affect the acceptability of drinking water, it is an essential element in human nutrition. Iron ranged from 0.01 to 0.05 mg/L in all the brands. All the brands complied with both the MS 699 (2004) and MS 560 (2004) iron guideline values of 1 mg/L for bottled water and 2 mg/L for natural mineral waters, respectively. However, the levels of iron were not sufficient to provide a mineral supplement to consumers necessary for the formation of the protein haemoglobin and cellular metabolism [37].

There were significant differences among the brands (ANOVA, *p* < 0.05) for both Ca and Mg. All the bottled water brands (B3-B12) registered Ca values (12.8–99.3 mg/L) below the MS 699 (2004) standard of 150 mg/L. Likewise, mineral brands B1 and B2 complied with the MS 560 (2004) *Ca* standard of 100 mg/L. There are no guideline values for both Ca and Mg in drinking water by WHO (2017). However, the taste threshold range of 100–300 mg/L is provided for Ca [37]. Brands B1 and B12 contained relatively higher Ca (99.3 mg/L, 64 mg/L) and Mg (40.8 mg/L, 48.6 mg/L) than the other brands. Drinking water containing high levels of both magnesium and sulphate (>250 mg/L each) may have a laxative effect, even though customers can adapt to such levels with time [37]. In this study, no brand scored Mg levels above 250 mg/L, hence the water could pose no laxative effects to consumers. A summary and classification of water hardness in line with selected classification are presented, as mean values, in Table 2 [36,44]. According to this classification, water hardness of the bottled water ranged from 64 to 416 mg/L CaCO_3_. More than half of the brands (58%, *n* = 12) were of ‘*moderately hard*’ class, followed by ‘*hard’* (25%) and ‘*very hard’* (17%). According to WHO [37], excess calcium intake is directed principally to people who are prone to milk alkali syndrome and hypercalcaemia. Furthermore, excess intake of magnesium salts may cause an impermanent adaptable change in bowel habits (diarrhoea), but infrequently leads to ‘*hypermagnesaemia’* in individuals with normal kidney function [44]. From our results, it is evident that frequent consumption of brands B1 and B12 that contained relatively high *Ca* and *Mg* concentrations may lead to such health implications.

Although calcium ingestion is significant at all ages, the need is higher during childhood, foetal growth, pregnancy, and lactation [45,46]. This is supported by previous epidemiological, animal, and clinical research works that reported existence of an inverse relation between *Ca* intake and occurrence of osteoporosis. For example, a diet or drinking water fortified with *Ca* may decrease the rate of age-related bone loss and hip fractures, particularly among adult females [45,47]. With reference to well-established dietary intakes (mg/day) [45], grown-up humans (19–50 years age range) need 1000 mg Ca^2+^, and 310–420 mg Mg^2+^ and 2400–3000 mg Na^+^. The water quality results were used to estimate the ingestion of *Mg*, *Ca* and *Na* by consumers from the bottled drinking water. Calculations were made using the minimum and maximum mean concentrations of the bottled drinking water with reference to the established dietary intakes and assuming ingestion of 2 L bottled water per day. The results showed that adult humans may fulfil only 2.6–20% of their Ca^2+^ dietary reference intake (DRI), 1–26% of their Mg^2+^ minimum DRI, and 1–19% of their Mg^2+^ maximum DRI. Further, low Na^+^ consumption of 0.2–3% of their Na^+^ minimum DRI and 0.2–2.5% of their Na^+^ maximum DRI were obtained. Amongst all the brands B1 scored the highest DRI for Ca^2+^, Mg^2+^ and Na^+^ ions. It is evident that a significant percentage of the population may be consuming insufficient Ca^2+^, Mg^2+^ and Na^+,^ which may have imperative health-related consequences [45,46].

### 3.2. Microbiological Quality and Their Health Implications

The *E. coli* bacteria are used as indicators of faecal contamination and to assess microbiological water quality [44]. The MS 699 [15], MS 560 [14] and WHO [37] recommend the colony counts of 0 per 100 mL sample for bottled water, natural mineral waters and any drinking waters, respectively. From our results no *E. coli* were detected, hence all the brands complied with the aforementioned standards. The bottled drinking water involved in this study was, therefore, of good quality at the time of sampling in as far as disease-causing microorganisms are concerned. Related microbiological results in which no *E. coli* were detected in bottled drinking water have been reported in Bolgatanga municipality (Ghana), Bangladesh and Argentina [43,48,49]. In contrast, total coliforms and *E. coli* were detected in only one of the sixteen brands of bottled drinking waters investigated in Nigeria [50]. Furthermore, in another related study on microbiological quality of mineral waters in Brazil, four brands out of five were reported to be contaminated with total coliforms, faecal coliforms, and *Pseudomonas aeruginosa* [10]. Investigation on some bottled drinking water brands marketed in Jaipur city India, Zimbabwe, Tokyo city in Japan, Trinidad and Hungary reported to contain either *P. aeruginosa*, Psychrophillic, *Enterococcus* spp., coliforms, *E. coli*, Penicillium, Acremonium, Cladosporium or Staphylococcal bacteria [51,52,53,54,55]. In this study, other microbiological indicators were not analysed due to financial and time constraints. Similar studies in the future may, therefore, consider analysing other microbiological indicators.

### 3.3. Brands and Chemical Composition Classification

#### 3.3.1. Hierarchical Cluster Analysis

**Figure 5:**
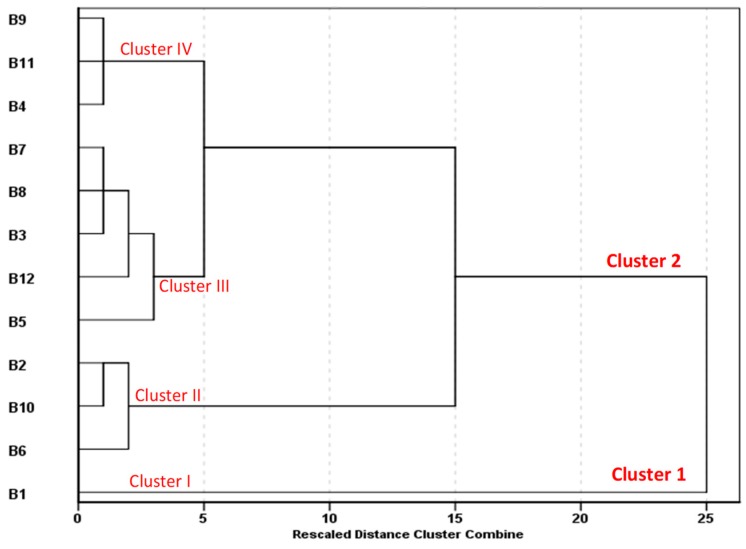
Hierarchical cluster analysis dendrogram showing clusters of 12 bottled water brands with respect to their chemical composition.

Figure 5 shows results of the hierarchical cluster analysis (HCA) used to detect groupings and homogeneity among the 12 bottled water brands. The brands were classified according to their chemical compositions. Homogeneity within clusters was based on Euclidean distance and Ward’s method was used to obtain hierarchical associations. At a rescaled distance cluster combined of 25, two groups were formed: Cluster 1 (B1) and Cluster 2 (B2–B12). From the results, it is evident that B1 had a unique chemical composition and this was supported by its water source, the natural spring. A rescaled distance cluster combined of five produced four sub-clusters, namely cluster I (B1), cluster II (B2, B6, B10), cluster III (B3, B5, B7, B8, B12) and cluster IV (B4, B9, B11). The cluster of B2 is closely associated with that of B1 and this is attributed to the fact that they were mineral water and spring brands. The concentrations of various water quality parameters in brands B4, B9 and B11 were very low, hence formed their own cluster. This was attributed to their combined treatment processes (Table 1) that probably removed most of the ions from the raw water.

#### 3.3.2. Principal Component Analysis (PCA)

A summary of PCA results is presented in Table 3. The 12 bottled water brands gave Bartlett’s test of sphericity 1898.73 and Kaiser-Meyer-Olkin (KMO) measure of sampling adequacy of 0.77, a value higher than the suggested KMO threshold 0.50–0.60 for PCA. Furthermore, high commonalities for all the measured parameters were obtained. Varimax normalisation procedure for eigenvector rotation resulted in three principal components (PC1, PC2, and PC3), which explained about 77.70% of the total variance. PC1 was associated with most of the parameters (pH, TDS, EC, *Na*, *Mg*, SO_4_^2−^, Cl^−^, alkalinity and F^−^), explaining about 51% of the total variance. In this component, strong positive loadings were obtained for TDS (0.99), EC (0.98), *Na* (0.83), *Mg* (0.76), Cl^−^ (0.92), alkalinity (0.97), and F^−^ (0.76). These results are consistent with correlation analysis in which significant correlations (r ≥ 0.50, *p* < 0.05) existed among these parameters. PC2 accounted for a total variance of 13.74% and had moderate to strong positive loadings on three parameters, namely *Ca* (0.73), *K* (0.52) and *Fe* (0.90). PC3 with a total variance of 12.95% explained two strong loadings for *K* (0.73) and NO_3_^−^ (−0.70). Previous studies employed multivariate tools, such as PCA and cluster analysis, for the classification of bottled drinking water reported similar findings [7,11,56]. 

### 3.4. Chemical Composition and Correlation Matrix

**Figure 6:**
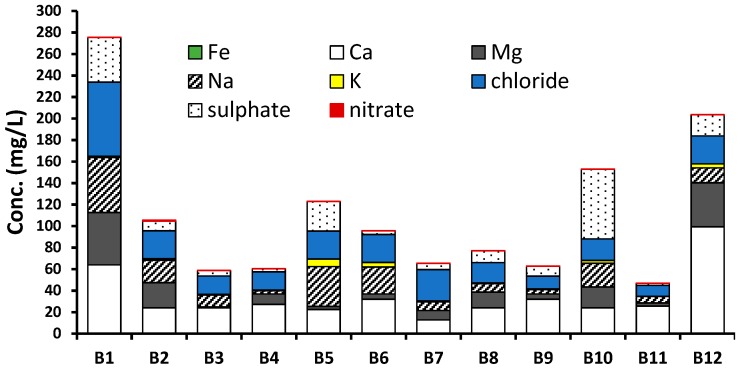
Mean concentrations and proportion of major ionic constituents (*Na*, *K*, *Fe*, *Ca*, *Mg*, Cl^−^, SO_4_^2−^, NO_3_^−^) in the water brands. Data for each brand are the mean values of the total samples (*n* = 30).

Figure 6 shows a graph on mean concentrations and proportion of major ionic constituents (*Na*, *K*, *Fe*, *Ca* and *Mg*, Cl^−^, SO_4_^2−^, NO_3_^−^) in all the water brands. Generally, brand B1 showed the highest ionic composition (275 mg/L) followed by B12 (203 mg/L) and B10 (152 mg/L). The lowest ionic composition was measured in B11 (46.90 mg/L). The highest ionic composition in B1 was attributed to its water source (natural mineral) and the treatment process (ozonation). The natural spring and mineral waters are reported to have high ionic content as their chemical compositions are usually controlled by many factors, including the chemistry of atmospheric precipitation, mineralogy of the rocks, the residence time of the groundwater in the aquifer, climate and topography [9,13]. The mineral content (*Na*, *K*, *Fe*, *Ca* and *Mg*) of the bottled waters varied significantly, and this is consistent with studies conducted with commercially available North American, European and Canadian bottled drinking waters [12,45]. Furthermore, comparable results in which mineral water contained relatively high ionic content were reported in five commercial brands of bottled water in Estonia [57]. Generally, the *Na*, *Ca*, *Mg*, Cl^−^, and SO_4_^2−^ were the major constituents in all the brands, and similar findings have been reported for bottled drinking water sold in Mwanza city, Tanzania [9]. The *Fe* and NO_3_^−^ were generally the least constituents in all the brands and this is consistent with similar studies elsewhere [9,16,17,43].

The correlation matrix (Pearson Correlations, r) for various water quality parameters in the bottled water brands are presented in Table 4. Most chemical constituents of the bottled water brands exhibited variations in correlations, and this was attributed to the differences in their water source and treatment processes. The pH showed positive correlation with *Na* (r = 0.63, *p* < 0.05), while turbidity correlated negatively with Cl^−^ (r = −0.58, *p* < 0.05). TDS and EC showed strong positive and significant correlations with following parameters: *Mg* (r = 0.70, *p* < 0.05), *Na* (r = 0.88, *p* < 0.01), Cl^−^ (r = 0.91, *p* < 0.01), SO_4_^2−^ (r = 0.59, *p* < 0.05), alkalinity (r = 0.98, *p* < 0.01), and F^−^ (r = 0.70, *p* < 0.05). The results are comparable with those reported for bottled drinking water in Mwanza City in Tanzania [9]. No significant correlations were obtained for potassium, nitrate and iron with the rest of the measured parameters, and this corresponds well to their chemical compositions which were low in all the bottled water brands.

### 3.5. Comparison between Analytical Results and Bottle Label Values

The number of water quality parameters reported and a comparison of the physico-chemical results with the claimed values are shown in Table 1; Table 5, respectively. Brands B6, B7, B10 and B11 registered pH values significantly higher than the claimed values. Four brands (B4, B7, B9 and B11) reported significantly lower TDS compared to the actual measured values. Conversely, two brands (B3 and B8) exaggerated their TDS values, while the actual measurements were apparently lower. Brand B2 had lower turbidity (0.40 NTU) of their bottled water than the actual measurement (1.10 NTU). Despite the need for turbidity to be included in the chemical composition of bottled water, most of the brands (92%, *n* = 12) did not report it. This is a clear and serious violation of MS 560 (2004) and MS 699 (2004) specifications that require accurate labelling of the chemical composition of any bottled water. One brand (B1) has a high value of *Ca,* while four brands (B2, B3, B4) have high values of *Mg* of their composition than the actual concentrations which were significantly lower.

Bottled water labelling errors—both undervaluing and exaggeration—were also noted in the rest of the water quality parameters (*Na*, *K*, Cl^−^, SO_4_^2−^ NO_3_^−^, F^−^ and *Fe*). Interestingly, most of the brands generally did not report turbidity, NO_3_^−^, alkalinity and *Fe* on their bottle labels. Brand B7 had the lowest number of water quality parameters (*pH*, *Ca*, Mg, Cl, and *F*) (46%, *n* = 13) indicated on its bottle labels. This was followed by brand B11 in which only seven water quality parameters (54%, *n* = 13) were labelled. Out of 13 parameters, brands B1, B3 and B4 had eight of them present on their labels. Furthermore, a fair number of the brands (38%, *n* = 13: B4, B5, B6, B10 and B12) did not report exact ranges or concentrations of the chemical parameters, they instead resorted to using ‘*greater than’* (>) or ‘*less than*’ (<) symbols. Despite being acceptable for TDS only for natural mineral waters–specification (MS 560; 2004), this tendency was being abused by most of the companies which perhaps wanted to conceal the other chemical compositions of their bottled water. This study also revealed that some brands (33%, *n* = 12) (Table 1) did not indicate the type and source of their water on the bottle labels. Furthermore, about half of the brands (50%, *n* = 12) concealed their treatment methods. This is worrisome as consumers and regulatory bodies are supposed to know such useful information.

Although brand B2 had its bottled water labelled with most of the water quality parameters, the majority of them (85%, *n* = 13) did not comply with our results. Additionally, the chemical contents for most of the parameters reported by B2 brand were significantly different (ANOVA, *p* < 0.05) from the actual values. This is a serious violation of the MS 560 (2004) guidelines and displayed negligence or lack of knowledge about the required chemical composition of drinking water. The study suspected the bottle labels were made based on previous analytical results, during which significant changes in water chemical composition may have occurred at the source. The irregularities between the measured and labelled values might be attributed to either typographic error or deliberate act to conceal the actual chemical composition of the bottled waters. Additionally, the variations in chemical compositions revealed in this study might have occurred due to changes in physical and chemical processes, such as (co)precipitation after exposure to light and storage of the bottles. The discrepancies in the chemical compositions found in this study and elsewhere [58] may also be attributed to accuracy, precision and detection limits of the analytical methods and instruments used by the bottled water companies. Despite such possible sources of error in chemical compositions and bottle labelling, companies selling the bottled water must prioritise the supply of safe water. This is also supported by the current Sustainable Development Goal (SDG) 6 target 6.1 where provision, access to safe and affordable drinking water is promoted among nations [59]. Dubious labelling and inconsistencies in the chemical composition of bottled drinking water have also been reported in Mwanza city (Tanzania), Turkey, Iran, Italy, Canada, and Egypt [9,12,16,17,18,58,60,61].

## 4. Conclusions and Recommendations

This study investigated the chemical composition of bottled drinking water sold in Lilongwe city, Malawi. Compliance with national and international drinking water standards and the potential health implications associated with consumption of the bottled drinking water brands were investigated. The results showed significant differences (ANOVA *p* < 0.05) in chemical compositions among the 12 brands. Ten brands did not comply with the MS 699 (2004) turbidity standard of 1 NTU. One brand had its mean pH (5.77) below the minimum MS 699 (2004) value of 6.5. The TDS, EC, *Ca*, *Mg*, *Na*, *K*, *Fe*, NO_3_^−^, Cl^−^, SO_4_^2−^, alkalinity and *E. coli* complied with the MS and WHO guidelines. Two mineral water brands failed to meet the MS 560 (2004), while four brands had the lowest F^−^ values. These brands required fluoridation to prevent dental caries in consumers. The two clusters obtained from the HCA depicted variations in water sources and treatment processes. Generally, the majority of brands (except two) complied with MS and WHO standards during the time of the study. One brand and two brands did not comply with pH and fluoride MS guidelines, respectively. Bottled water labelling errors and discrepancies—both undervaluing and exaggeration—were noted for *Na*, *K*, Cl^−^, SO_4_^2−^ NO_3_^−^, F^−^ and *Fe*. While one brand seriously violated the chemical labelling obligation, all the brands showed either negligence or lack of knowledge about the required chemical composition. Consequently, the following recommendations were drawn (i) strict bottle labelling must be enforced and inclusion of microbiological water quality data must be considered, (ii) considering non-compliance on turbidity and F^−^ for some brands, regulatory bodies must intensify their activities to ensure total compliance and to safeguard safety of consumers, (iii) besides *E. coli*, future studies must investigate other equally important microorganisms in bottled drinking water, (iv) fluoridation and mineral fortification would be possible remedies for the brands that had the lowest fluoride and chemical content of *Mg*, *Ca* and *Na*, (v) related studies must be conducted on bottled water with large sample sizes collected from the other cities and towns in Malawi, and the regulations must consider enforcement of Hazard Analysis and Critical Control Points (HACCP) to ensure safe limits of bottled drinking water at all stages of production.

## Figures and Tables

**Table 1:** Brand water type, source and treatment processes used.

Brand Code	Type and Source	Treatment Method	Percentage of Water Quality Parameters Reported (*n* = 13)
B1	Natural spring	Ozonation	62% (Ca, Mg, Na, K, Fe, Cl^−^, SO_4_^2−^, F^−^)
B2	Non-carbonated natural mineral	NA	100% (pH, TDS, turbidity, alkalinity, Ca, Mg, Na, K, Fe, Cl^−^, SO_4_^2−^, NO_3_^−^, F^−^)
B3	Purified water	NA	62% (pH, TDS, Ca, Mg, Na, K, Cl^−^, SO_4_^2−^)
B4	Ultra-pure distilled water	SCF, RO, UV-I and Ozonation	62% (pH, TDS, Ca, Mg, Na, K, Cl^−^, SO_4_^2−^)
B5	Purified still	NA	77% (pH, TDS, alkalinity, Ca, Mg, Na, K, Cl^−^, SO_4_^2−^, F^−^)
B6	Premium still	NA	85% (pH, TDS, alkalinity, Ca, Mg, Na, K, Cl^−^, SO_4_^2−^, NO_3_^−^, F^−^)
B7	NA	RO, UV-I	46% (pH, TDS, Ca, Mg, Cl^−^, F^−^)
B8	Purified still	UV, RO, Ozonation	69% (pH, TDS, Ca, Mg, Na, K, Cl^−^, SO_4_^2−^, F^−^)
B9	Na	Filtration, RO, UV-I	77% (pH, TDS, alkalinity, Ca, Mg, Na, K, Cl^−^, SO_4_^2−^, F^−^)
B10	NA	NA	85% (pH, alkalinity, Ca, Mg, Na, K, Fe, Cl^−^, SO_4_^2−^, NO_3_^−^, F^−^)
B11	NA	RO	54% (pH, TDS, Ca, K, Cl^−^, SO_4_^2−^, F^−^)
B12	Purified still	NA	77% (pH, TDS, alkalinity, Ca, Mg, Na, K, Cl^−^, SO_4_^2−^, F^−^)

NA: data not available. RO: Reverse Osmosis, SCF: sand column filtration. UV-I: ultraviolet irradiation.

**Table 2:** Classification of the water hardness compared to the current study *.

Conc. Range (mg/L CaCO_3_)	Hardness Classification	Comparison with This Study: Brand (Hardness, mg/L CaCO_3_)
**<60**	Soft	-
**60–120**	Moderately hard	**B3** (64), **B4** (108), **B5** (68), **B6** (100), **B7** (68), **B9** (100), **B11** (76)
**120–180**	Hard	**B2** (156), **B8** (120), **B10** (140)
**>180**	very hard	**B1** (359), **B12** (416)

Conc.: concentration. -: not applicable. * Classification based on WHO [44] and Lenntech [36].

**Table 3:** Water quality parameters for the 12 bottled water brands.

Parameter	Principal Component *		
	PC1	PC2	PC3
pH	**0.55**	−0.10	**0.60**
TDS	**0.99**	0	0.06
EC	**0.98**	0	0.06
*Ca*	0.35	**0.73**	−0.10
*Mg*	**0.76**	0.40	−0.24
*Na*	**0.83**	0.19	0.42
*K*	0.05	**0.52**	**0.73**
Cl^−^	**0.92**	0.11	0.11
SO_4_^2−^	**0.59**	−0.08	0.33
NO_3_^−^	0.08	0.14	**−0.70**
ALK	**0.97**	0	−0.01
F^−^	**0.76**	0.28	−0.18
*Fe*	−0.13	**0.90**	0.04
Eigen value	6.63	1.79	1.68
% of variance	51.01	13.74	12.95
Cumulative %	51.01	64.75	77.70

ALK: alkalinity; PC: principal component. * Sample size (*n*) = 360. Bold-faced values indicate the highest loadings (≥0.50).

**Table 4:** Correlation matrix for the physico-chemical water quality parameters.

	pH	Turbidity	TDS	EC	*Ca*	*Mg*	*Na*	*K*	Cl^−^	SO_4_^2−^	NO_3_^−^	ALK	F^−^	*Fe*
pH	1													
Turbidity	−0.15	1												
TDS	0.54	−0.30	1											
EC	0.54	−0.29	0.99 **	1										
*Ca*	−0.05	−0.26	0.32	0.32	1									
*Mg*	0.16	−0.36	0.70 *	0.70 *	0.78 **	1								
*Na*	0.63 *	−0.37	0.88 **	0.88 **	0.27	0.51	1							
*K*	0.29	−0.22	0.10	0.10	0.18	−0.01	0.53	1						
Cl^−^	0.56	−0.58 *	0.91 **	0.91 **	0.42	0.74 **	0.82 **	0.12	1					
SO_4_^2−^	0.29	0.15	0.59 *	0.59 *	0.21	0.50	0.59 *	0.32	0.42	1				
NO_3_^−^	−0.39	−0.15	0.08	0.08	−0.16	0.10	−0.03	−0.20	−0.07	−0.14	1			
ALK	0.55	−0.34	0.98 **	0.98 **	0.28	0.64 *	0.84 **	0.05	0.91 **	0.44	0.13	1		
F^−^	0.47	−0.28	0.70 *	0.70 *	0.40	0.65 *	0.54	0.06	0.62 *	0.28	0.26	0.76 **	1	
*Fe*	0.01	−0.45	−0.11	−0.11	0.43	0.13	0.13	0.46	−0.01	−0.28	0.22	−0.07	0.19	1

* Correlation is significant at alpha (α) *=* **0.05** level (2-tailed). ** Correlation is significant at alpha (α) *=* **0.01** level (2-tailed). ALK: alkalinity.

**Table 5:** Physico-chemical results (mean values) compared to claimed values.

Brand	Item	pH	TDS	Turb	*Ca*	*Mg*	*Na*	*K*	Cl^−^	SO_4_^2−^	NO_3_^−^	ALK	F^−^	*Fe*
**B1**	CL	NA	NA	NA	100	35	50	2.0	100	61.0	NA	NA	0.40	NA
	AL	8.11	585.4	0.6	64 **	48.6 *	51	1.2 **	69 **	41.5 **	0.032	320	0.86 *	0.018
**B2**	CL	6.99	210	0.4	19	69	11	8.0	17	15	0.08	18	0.005	0.01
	AL	6.89	212	1.1 *	24 *	23.3 **	21 *	1.3 **	26 *	8.99 **	0.865 *	140 *	0.79 *	0.04 *
**B3**	CL	7.20	200	NA	0.55	7.4	11.4	5.0	12.9	84	NA	NA	NA	NA
	AL	7.67	73.8 **	2.0	24 *	1.0 **	**11.0**	0.6 **	17 *	5.01 **	0.033	60	0.5	0.037
**B4**	CL	6.6	5	NA	>20	>20	>30	>10	>5	>250	NA	NA	NA	NA
	AL	6.65	12.3 *	2.0	27.2	9.7 **	3 **	0.6 **	17	2.69 **	0.013	20	0.28	0.024
**B5**	CL	6.5	<100	NA	<150	<70	<200	<50	<200	<400	NA	<200	<1.0	NA
	AL	7.61	126.2	1.0	22.4	2.9	37	7.1	26	27.44	0.012	60	0.21	0.042
**B6**	CL	6.0	<200	NA	<50	<20	<30	<10	<20	<80	<0.05	<85	<0.7	NA
	AL	7.87 *	170.8	2.0	32	4.9	25	4.3	26 *	3.38	0.012	**136**	0.82 *	0.026
**B7**	CL	6.80	5	NA	2.48	0.031	NA	NA	0.19	NA	NA	NA	0.002	NA
	AL	7.55 *	55.8 *	0.5	12.8 *	8.7 *	8.0	1.0	29 *	5.82	0.033	48	0.39 *	0.022
**B8**	CL	7.80	110	NA	23	11	9.0	18	18	12.0	NA	NA	<0.01	NA
	AL	7.40	64.1 **	2.0	24	14.6 *	8.0	0.5 **	19	10.9 **	0.062	44	0.52 *	0.019
**B9**	CL	5.77	7	NA	16	0.97	5.1	0.2	10.5	7.96	NA	76	0.34	NA
	AL	5.77	28.3 *	2.0	32 *	4.9 *	4.0 **	0.6 *	12	9.16 *	0.075	28 **	0.23	0.017
**B10**	CL	6.0	NA	NA	<50	<20	<30	<10	<20	<80	<0.05	<85	<0.7	<0.1
	AL	7.43 *	213.8	3.9	24	19.4	22	2.7	20	64.69	0.063	92 *	0.58	0.006
**B11**	CL	6.58	5	NA	2.48	NA	NA	0.003	0.019	0.003	NA	NA	0.002	NA
	AL	7.16 *	31.3 *	4.2	25.6 *	3	6.0	0.2 *	10 *	2.06 *	0.064	28	0.24 *	0.019
**B12**	CL	6.5–8.5	<100	NA	<150	<70	<200	<50	<200	<400	NA	<200	<1.0	NA
	AL	6.85	79.22	1.2	99.2	40.8	14	3.7	26	19.57	0.024	40	0.63	0.053

CL: claimed value: AL: Actual values. NA: data not available. Turb: turbidity (in NTU). ALK: alkalinity as CaCO_3._ Units: mg/L except for pH and turbidity. * Significantly above claimed value. ** Significantly below claimed value. Significance level (*a*) of 0.05 was used to determine the statistical differences for the values denoted with superscripts (* and **).
